# Goalsetting is Mindsetting: Guided Reflection on Life Goals Taps Into the Plasticity of Motivational Mindsets

**DOI:** 10.1177/00332941231180813

**Published:** 2023-06-06

**Authors:** Job Hudig, Ad W. A. Scheepers, Michaéla C. Schippers, Guus Smeets

**Affiliations:** 168103 Erasmus School of Social and Behavioral Sciences, Erasmus University, Rotterdam, The Netherlands; 84857 Rotterdam School of Management, Erasmus University, Rotterdam, The Netherlands; 84857 Rotterdam School of Management, Erasmus University, Rotterdam, The Netherlands; 168103 Erasmus School of Social and Behavioral Sciences, Erasmus University, Rotterdam, The Netherlands

**Keywords:** motivation, higher education students, personal goals, deductive coding, student success, reasons for attending university

## Abstract

The working mechanism of an effective online lifegoal-setting intervention was recently proposed by means of the motivational mindset model (MMM). The MMM contains four types of mindset profiles (high-impact, low-impact, social-impact, and self-impact) based on multiple, co-occurring motives that students hold for studying. The present paper aims to qualitatively investigate the mechanism and explores whether the goal-setting intervention fosters a favorable change in mindset. To this end, a deductive content analysis was used to examine the life goal motives in the written goal-setting essays of 48 first-year university students (33% female; 8.3% ethnic minority; M_age_ = 19.5, age range 17–30 years). Life goal motives were coded according to four dimensions along two distinctions (self-oriented versus self-transcendent, and intrinsic versus extrinsic) and analyses were focused on comparisons between changed and stable mindsets. Results show that students who changed from a low-impact mindset to a social-impact mindset expressed intrinsic self-oriented and intrinsic self-transcendent motives to a similar extent as stable social-impact mindset students. This pattern indicates that the positive change in mindset already occurred during the reflection assignment and substantiates the proposed mechanism of the goal-setting intervention. The implications of the findings are discussed as well as directions for future research.

## Introduction

The first year of university is a critical time for students to complete their bachelor’s degree (van der Zanden et al., 2018; Willcoxson et al., 2011). Many interventions are therefore focused on the first year to help students in their educational process (Harackiewicz & Priniski, 2018). An intervention that has been shown to promote first-year study success is an online goal-setting intervention. This intervention entails a reflection assignment where freshmen spend four to six hours writing about themselves, their best-possible-selves, and their personal life goals. Studies on the effects of the goal-setting intervention have shown promising results (Morisano et al., 2010; Schippers et al., 2015, 2020). Study success rates increased by 22% and especially male students and students with an ethnic minority background benefitted from the guided reflection on life goals (Schippers et al., 2015). Little is known, however, about the psychological processes that drive these positive effects. Knowing when, how, why and for which students the goal-setting intervention generally works is key to be able to help more students boost their academic performance. An important potential mechanism might work via the motivational mindset of students (Hudig et al., 2022).

Motivational mindsets relate to different combinations of co-occurring motives for studying, or reasons why students attend university. These motives have shown to strongly influence students’ performance and academic integration (Dennis et al., 2005; Kennett et al., 2013; Wang et al., 2009). The motivational mindset comprises three dimensions of study motives: (1) intrinsic self-transcendent motives (i.e., studying to make the world a better place), (2) intrinsic self-oriented motives (i.e., studying because it is personally interesting and enjoyable), and (3) extrinsic self-oriented motives (i.e., studying to acquire a separable outcome, such as money or making new friends). Prior research showed that these motivational dimensions were independently related to academic functioning and wellbeing (Yeager et al., 2014). Hudig et al. (2020, 2021) tested the joint effects of the dimensions to (a) better understand differences in study success and wellbeing, and (b) understand why interventions are effective for some first-year students and not for others. Accordingly, they developed their motivational mindset model (MMM) which discerns four types of mindset profiles, namely high-impact, low-impact, social-impact, and self-impact.

High-impact mindset students generally possess high levels of all motivational dimensions, whereas low-impact mindset seem to go to university for shallower extrinsic reasons. Social-impact mindset students are primarily intrinsically driven, while self-impact mindset students hold a strong extrinsic orientation that also includes intrinsic self-oriented reasons for studying. The mindsets are not fixed, however. Hudig and colleagues (2022) found that more than half of the students changed mindset over the course of the first year which implies a plasticity of the motivational mindsets. This mindset churn was particularly relevant for low-impact mindset students in the context of the goal-setting intervention. Findings indicated that the sense of purpose increased after goalsetting specifically for students who changed from a low-impact mindset to a social-impact mindset. This pattern provided preliminary support that the goal-setting intervention is a purpose-fostering intervention for students entering higher education with a low-impact mindset. Yet the extent to which mindset churn is a result of the goal-setting intervention or a more spontaneous event requires further investigation.

The current study therefore adopts a qualitative approach to further explore the impact of the goal-setting intervention on the motivational mindset of students. Specifically, a content analysis was used to examine the written goal-setting essays on the *motives* that students have for their life goals (for a related discussion of goal content, see Vansteenkiste et al., 2004). While the quality and quantity of the written goal-achievement plans have been studied before via content analysis (Schippers et al., 2020), an extensive qualitative analysis on the content of the goal-setting intervention in the context of changing motivational mindsets has not yet been conducted. Prior research showed that the quantity and quality of the goal achievement plans are positively related to study success (Schippers et al., 2020). The extent to which the writing is related to a mindset churn is key to our understanding of motivational stability and change after students performed the goal-setting intervention. The exploration of the reflective writings may also provide more insight into characteristic differences across the four mindsets which enriches the MMM. Importantly, this paper has practical relevance as it aims to further our understanding of the goal-setting intervention in order to support more students who need it in their academic and professional careers.

### Theoretical background

The MMM consists of four motivational mindsets which are distinct in several ways (Hudig et al., 2020). High-impact mindset students are characterized by high levels of intrinsic self-transcendent motives, intrinsic self-oriented motives, and extrinsic motives. In other words, they aim to positively impact all aspects of their lives through their university studies. For instance, they want to be successful in earning high grades and doing well financially, but also focus on personal growth and finding purposeful work. Low-impact mindset students on the other hand are characterized by low levels of intrinsic self-transcendent and self-oriented motives, and moderate extrinsic motives. These students merely go to university because it is expected of them. Besides having an active social life and earning money in the future, they generally have no clear perspective on why they go to university. Social-impact mindset students are characterized by moderate to high levels of intrinsic self-transcendent and self-oriented motives, and low levels of extrinsic motives. These students are primarily eager to learn and this drive is grounded in wanting to have a positive effect on people and communities through their studies. Finally, self-impact mindset students are characterized by high levels of extrinsic motives, moderate levels of intrinsic self-oriented motives, and low levels of intrinsic self-transcendent motives. These students are particularly driven to achieve personal success. They view their university studies as the “doorway” to this success and tend to be strongly money- and career-oriented (see also Table 1 for a summary of the mindset profiles and their motivational dimensions) (Hudig et al., 2020).

**Table 1.** Summary of the motivational mindset profiles according to their level of motives for studying.

	Motivational mindset			
	High-impact	Social-impact	Self-impact	Low-impact
Intrinsic, self-transcendent motives	*high*	*moderate-high*	*low*	*low*
Intrinsic, self-oriented motives	*high*	*moderate-high*	*moderate*	*low*
Extrinsic, self-oriented motives	*high*	*low*	*high*	*moderate*

Most prominent churn between mindsets: high-to-social, social-to-high, low-to-social, and self-to-low (Hudig et al., 2022).

Follow-up research on the MMM showed that the four mindsets differ meaningfully in sense of purpose and study engagement (Hudig et al., 2021). Sense of purpose is the extent to which a student perceives one has personally meaningful long-term goals and a sense of direction in life (Ryff, 1989). Purpose is related to several important developmental outcomes (for reviews see: Ryff, 2014; Schippers & Ziegler, 2019) and is one of the central dimensions of mental wellbeing (Dahl et al., 2020). Study engagement is a positive and fulfilling study-related state of mind that comprises levels of energy, dedication, and concentration while studying (Schaufeli et al., 2002), and has been related to both wellbeing and academic performance (Upadyaya & Salmela-Aro, 2013; Widlund et al., 2018). In terms of the MMM, a study among 662 first-year students showed that high-impact mindset and social-impact mindset students were most similar and showed the most optimal pattern (i.e., highest levels) in purpose and engagement. Self-impact mindset students had a weaker sense of purpose compared to the high-impact and social-impact mindset, while these students showed to have equal engagement levels as the social-impact mindset students. Low-impact mindset students demonstrated the least optimal pattern in the two dimensions of wellbeing: the weakest sense of purpose and lowest level of study engagement (Hudig et al., 2021). Regarding academic performance, research showed that students who maintain a low-impact mindset throughout the first year can be just as successful as the other three motivational mindsets. Students who adopt a low-impact mindset during the study program, however, have a higher risk of dropping out (Hudig et al., 2022).

In the context of the goal-setting intervention, Hudig et al. (2022) proposed that the goal-setting intervention impacts students’ sense of purpose. Purpose, in turn, has been positively related to engagement (Hudig et al., 2021) and the positive connection between study engagement and academic performance has been shown extensively (e.g., Ouweneel et al., 2011). Hence, the authors explored whether the constellation of these constructs would fit into a potential working mechanism of the goal-setting intervention. Specifically, Hudig et al. (2022) examined the extent to which the goal-setting intervention had a different impact on the pre-intervention levels of purpose and study engagement of the various mindsets. In the study, *stable* mindsets and *changed* mindsets were identified.

Interestingly, students with an initial low-impact mindset that changed to a social-impact mindset over the course of eight months after participating in the goal-setting intervention, were the only group who reported a significant increase in purpose (Hudig et al., 2022). This group also showed a steady upward trend in study engagement while a decline of engagement was demonstrated in the other part of the cohort. Based on this pattern of findings, the authors speculated that the strategic goalsetting may have differed for the different mindset groups. They suggested that via the setting of goals in the intervention, a sense of purpose in unstable low-impact mindset students may have been fostered, since these are students who did not really consider or explicate their reasons for studying before going to university. The goal-setting intervention may therefore be especially useful for these kind of students (cf. Schippers et al., 2020). Possibly, as a result of intensive reflection, these students are helped to promote a shift in thinking toward intrinsic self-oriented and intrinsic self-transcendent motives. This is also nudged by the intervention, as students are for instance explicitly asked to reflect on their values and passions and are urged to think about activities and to make (career related) choices that energize them (Schippers & Ziegler, 2019). The enhanced feelings of purpose in combination with an expanded motivational mindset then yields a buffering effect of engagement, indicating that the engagement levels were protected from a general decline during the first academic year (Hudig et al., 2022). This process would explain how the goal-setting intervention is especially potent to boost the study success of academically less-motivated students.

Notably, prior research also showed a mindset churn where self-impact mindset students at the beginning of their studies prominently changed to a low-impact mindset. It seemed that some students somehow experienced a loss of motivation and changed to a less optimal mindset in terms of study motivation. These students demonstrated strong declines in purpose and engagement over the course of the first year and the authors speculated that the goal-setting intervention may also have a ‘negative’ influence on this specific subgroup of students (Hudig et al., 2022). Students might find out that the study program does not match their life goals or that they have other (less favorable) motives than previously thought.

Because the mindset was measured at the beginning and the end of the academic year, we aimed to further investigate whether participating in the goal-setting intervention induced these mindset changes.

### Present study

The present study builds on the findings by Hudig et al. (2022) and aims to contribute to understanding the working mechanism of the goal-setting intervention. The central **Research Question** is: *To what extent are the goal-setting essays of students indicative for a mindset change?* In order to investigate this research question, we will examine the essays of two sets of mindset groups. The first set contains the four stable mindsets with the same motives at baseline and follow-up. We explore the life goal motives mentioned in the essays and compare the constellations of these motives across the four stable motivational mindsets. Further, we examine a second set of student essays which includes the two most relevant changed mindset groups: low-to-social and self-to-low. Again, we observe the constellation of motives in the goal-setting essays of these students and examine the extent to which the motives in the essays correspond to the mindset at baseline or follow-up. To this end, the stable motivational mindsets can be used as a benchmark to examine the extent of correspondence between the changed mindset and the stable mindset. We had no a priori hypotheses. But, if the constellation of motives in the essays of the changed mindset corresponds more with the characteristics of the stable mindset at follow-up than at baseline, that would indicate a change of mindset already occurring during the reflection assignment.

The present paper investigates the motives of students along the three motivational dimensions in line with studies by Hudig et al. (2020, 2021 & 2022) and Yeager et al. (2014). The existence of a fourth dimension - extrinsic self-transcendent motives - has been noted among adolescents in earlier qualitative work by Yeager and Bundick (2009) but was omitted from the quantitative survey studies. We decided to involve the extrinsic self-transcendent dimension considering the exploratory nature of our study. Definitions of the motivational dimensions were adapted from the literature and used as predefined categories to examine the student essays. Motives are intrinsic, self-oriented when the reason is inherently present in the goal or in the activity of striving towards the goal. Examples of these motives are learning, self-development, challenge, enjoyment, and getting energized from goal-striving (Kennett et al., 2011; Ryan & Deci, 2000). Motives are intrinsic, self-transcendent when the reason for the goal pertains to helping others (Yeager et al., 2012). Examples of such motives are doing something small each day as a gesture of kindness or wanting to do work that makes the world a better place. Motives are extrinsic, self-oriented when the reason for the goal concerns a separable outcome or reward when achieving the goal. Examples of these motives are money, status, professional success, material possessions, high grades, and a diploma (Kennett et al., 2011; Ryan & Deci, 2000). Based on the literature, self-pride was not regarded as an extrinsic motive but making others proud (e.g., parents) was declared as such (de Hooge & van Osch, 2021). Finally, motives for a goal are extrinsic, self-transcendent when the reason concerns a separable outcome to use in helping others. Examples of these motives entail acquiring money to support a charity or achieving status to be a role model. To narrow the scope of the present study and in line with the characteristics of the motivational mindset, we focus the analysis of the mindset comparisons on the *study- and work-related* motives mentioned in the student essays.

## Method

### Sample

The present study was part of a larger data collection effort and was conducted as a qualitative follow-up study on the effectiveness of the goal-setting intervention. Participants in the longitudinal study (*n* = 328) by Hudig et al. (2022) wrote a goal-setting essay and were classified into the motivational mindsets using the mindset classification tool (MCT) (for more details on the development and validity of the MCT, see Hudig et al., 2020, 2021). For the purpose of the current study, a subset sample of student essays was extracted from the quantitative study by Hudig et al. (2022) following the exact same relevant mindset groups. The two changed mindset groups were selected accordingly: the low-to-social-impact mindset with *n* = 9 and the self-to-low-impact mindset with *n* = 15. Subsequently, a set of 24 student essays was randomly selected from the stable mindset groups to match the number of student essays of the changed mindsets. Specifically, six student essays were withdrawn from each of the four motivational mindsets and concluded a total sample for this study of *n* = 48.

The 48 selected participants were all first-year bachelor students enrolled in the business administration program of a university in the Netherlands (academic year 2018–2019). Age ranged from 17 to 30 (M = 19.54, SD = 1.82), 33% were female, and 8.3% were non-Western ethnic minority students. The total sample of student essays closely approximated the survey sample conducted by Hudig et al. (2022) in terms of gender and ethnicity. The changed and stable mindsets were also compared in these demographic characteristics. The stable mindset group comprised 42% female students versus 21% in the changed mindset group. Regarding ethnicity, 4% in the stable mindset group had a non-Western ethnic background while this proportion was 13% in the changed mindset group. All 48 students had provided explicit consent for their participation in the research and the processing of their data.

### Procedure

**Figure 1.**
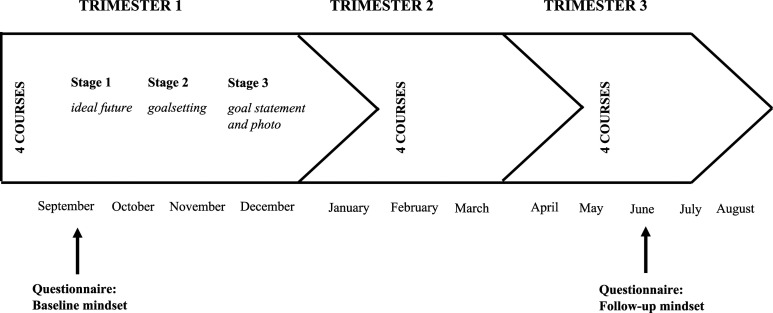
Study timeline goal-setting intervention (adapted from Hudig et al., 2022).

The three-stage online goal-setting intervention was part of a mandatory introductory course on Managerial Skills in the first year of the study program. Stage 1 of the intervention guides students through several open-ended questions concerning their current and future personal lives. Students end this stage with a free-writing exercise on the ideal future self. In Stage 2 students build on their reflections of Stage 1 by setting six to eight life goals and writing extensively through various guided questions about these future aspirations. Stage 3 concerns commitment in which students make their personal goal statement (i.e., “I WILL statement”) and have their portrait photo taken at a professional photoshoot (for an elaborate description of the goal-setting intervention see Schippers et al., 2015). Completion of Stage 1 and Stage 2 was mandatory to earn course credits. Stage 1 had to be finished three weeks into the academic year; Stage 2 two weeks later; and Stage 3 was scheduled after completing Stages 1 and 2 before the end of the first trimester (see Figure 1 for an overview of the study). Stage 1 and 2 were designed in Qualtrics and participation took place online at students’ location of choice.

In addition to the student data derived from the writing exercises, data on the motivational mindsets were collected in earlier research through online questionnaires (Hudig et al., 2022). The first questionnaire was completed by students prior to the goal-setting intervention at the start of the academic year (baseline). The second mindset measurement was completed towards the end of the regular academic year (8 months later; follow-up). Data on gender, ethnicity, and age were collected using university transcripts after the first year had finished. Students provided explicit informed consent to participate in the research prior to filling out the first online questionnaire. Tutors informed students about the purpose of the research and that participation in the research was voluntary. They notified students that they could withdraw their data from the research at any time and that their data would be treated confidentially. All methods were carried out in approval of the research school’s Internal Review Board and the research was compliant with the General Data Protection Regulation (GDPR).

### Measures

*Life goal motives* were derived from three parts of the goal-setting intervention (see the rationale for this decision in the next section): (1) future career, (2) ideal future, and (3) goal importance. In the ‘future career’ part of the goal-setting intervention, students were asked to think about their studies, education and/or career. The question focused mainly on what they regarded important in a job. In the ‘ideal future’ part of the goal-setting intervention, students were asked to think about what they would aspire to in an ideal world without obstacles. They were encouraged to write about their ideal future freely for 15 minutes. In the ‘goal importance’ part of the goal-setting intervention, students were asked to describe for each goal why this goal was so important to them. Both the ‘future career’ and ‘ideal future’ were part of Stage 1 of the intervention, while ‘goal importance’ was part of Stage 2 of the intervention.

### Analysis

Our qualitative study was conducted following a directed content analysis (Hsieh & Shannon, 2005). This content analysis entails a deductive coding process in which theoretically-driven codes are used. The codes in the current study were based on definitions of the four motivational dimensions provided earlier in the Introduction section of the paper. As a preliminary step, the first author got familiarized with the student essay data and conducted a pilot with three student essays to check whether the dimensions were formulated sufficiently.^1^ A structured categorization matrix was developed to assess each essay accordingly (Elo & Kyngäs, 2008). Authors 1, 2 and 4 conducted a second pilot in which they independently coded another set of three student essays using the coding matrix.^2^ The matrix was altered and refined following the discussion after this second pilot. Moreover, the pilot led to the decision to focus on three specific sections of the goal-setting intervention in which students appeared to formulate the reasons for their life goals, namely, the ideal future, the future career, and the six to eight descriptions of goal importance. Ultimately, the matrix contained six columns: four for each motivational dimension, one when no clear motive was mentioned (i.e., N/A), and one where the coder could not decide (i.e., indecision). Additionally, the matrix had eight to ten rows for the relevant intervention sections.

All the data (written essays) in the three sections of each essay were subsequently reviewed for content by three coders: the first author, second author and a senior researcher who was independent from the research study and an expert in the field of educational psychology. The 48 essays were coded twofold: the first author coded all 48 essays, the second author 24 essays and the senior researcher coded the other 24 essays. The essays were randomly allocated to coder 2 and 3, blinded in SPSS (to mask the mindsets for all three coders), and coded independently in the Excel program. Coders extracted the sentences (i.e., *coding references*) from each essay in which students referred to a goal motive relevant for our study. After all the data was coded, the final work file with coding references was established by means of three rounds of calibration between coders.

A first cycle of calibration was conducted in which each undecided goal motive was discussed for every participant. Trustworthiness of the coding process was based on consensus-building in a collaborative and iterative manner (Elo & Kyngäs, 2008; Hill et al., 1997). In addition, an indication of trustworthiness was established after the coding and first cycle of calibration were finalized by calculating the intercoder agreement between coders for each of the four dimensions using a pooled Kappa coefficient (de Vries et al., 2008). Agreement was acceptable with kappa’s between 0.60 and 0.80 (Altman, 1991) for the following codes: intrinsic self-transcendent (k = 0.72, percentage agreement = 96%), extrinsic self-oriented (k = 0.65, percentage agreement = 83%), and extrinsic self-transcendent (k = 0.79, percentage agreement = 98%). A low kappa of 0.37 and 72% percent agreement was reached for the intrinsic self-oriented dimension which required scrutiny in the next calibration cycle.

A second cycle of calibration was then scheduled with the three coders to create as much uniformity in the coding as possible. The first author worked through the coding and derived all the incongruent coding references between coders. Diligence was warranted in the intrinsic self-oriented dimension considering the relatively low agreement between coders. Nearly all incongruence was identified in one theme: positive emotions. Coder 1 versus coders 2 and 3 varied in their judgment of positive emotions as an intrinsic, self-oriented motive. Coders agreed in accordance with the literature that positive emotions are a related yet different construct from intrinsic motives (Løvoll et al., 2017). Positive emotions can indicate intrinsic motivation, and even facilitate intrinsic motivation, but are not the intrinsic motive itself. Cycle 2 contained several meetings to finalize the qualitative dataset. From here one last calibration session was conducted with the first and second author to filter out all the study- and work-related motives. A final workfile was created including all coding references and the integration of each motivational mindset membership. To help identify patterns in the qualitative data, the frequency of coding references was used (Maxwell, 2010). This allowed to discern the extent of correspondence across mindsets. Finally, a typical student for each motivational mindset was selected and descriptions were created to enrich the meaning and characteristics of the MMM. All names used in these descriptions are pseudonyms.

## Results

The content analysis of 48 student essays resulted in a total of 415 coding references. Of the total references in the coding, 209 references (50.4%) were derived from Stage 1 of the goal-setting intervention and 206 references (49.6%) from Stage 2. Regarding the motivational dimensions, 49% of coding references were intrinsic self-oriented, 40% extrinsic self-oriented, 7% intrinsic self-transcendent, and 4% extrinsic self-transcendent. The proportion of students who simultaneously had intrinsic and extrinsic motives related to their studies was 94% (45 of 48 students). Two students in the low-to-social group reported only intrinsic motives, and one student in the self-to-low group mentioned only extrinsic motives. Notably, 86% of coding references pertaining to the self-transcendent motives were provided by students in Stage 1 of the goal-setting intervention.

### Stable mindsets

**Figure 2.**
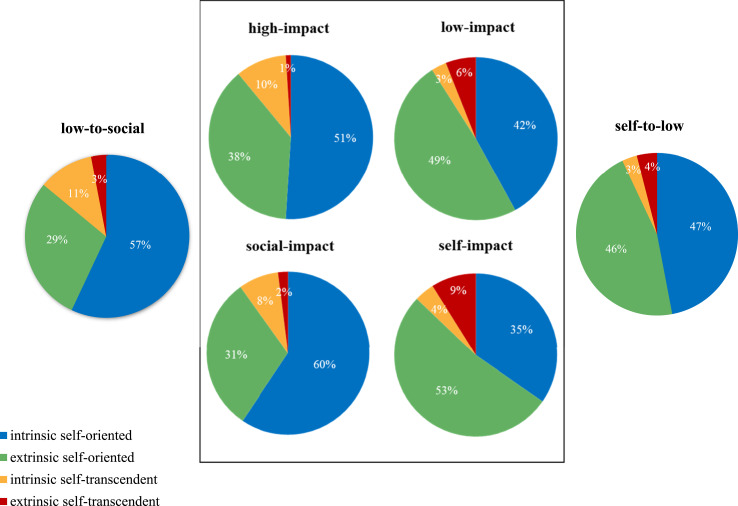
Coding references within stable mindsets (inside middle square) and changed mindsets (outside middle square) across the four motivational dimensions.

Table 2 and Figure 2 present the proportion of coding references *within stable motivational mindsets* across the four motivational dimensions. The social-impact mindset (60%) and high-impact mindset (51%) contained a considerably larger proportion of intrinsic self-oriented motives than the low-impact mindset (42%) and self-impact mindset (35%). In terms of extrinsic self-oriented motives, the distribution was inverted: students with a self-impact mindset (53%) and low-impact mindset (49%) referred more towards these types of reasons compared to the high-impact mindset (38%) and social-impact mindset (31%). Different patterns among stable mindsets also emerged in the two self-transcendent dimensions. Social-impact mindset (8%) and high-impact mindset (10%) had higher proportions of intrinsic self-transcendent motives compared to the self-impact mindset (4%) and low-impact mindset (3%). Extrinsic self-transcendent motives coding was more frequent among the self-impact mindset (9%) and low-impact mindset (6%) compared to the social-impact mindset (2%) and high-impact mindset (1%). Overall, a relatively small proportion of self-transcendent motives was reflected in the student essays. Of the 201 references coded among stable mindsets, 11% was beyond-the-self oriented (i.e., 22 references) of which 6% was intrinsic and 5% extrinsic. Nonetheless, 15 of 24 stable mindset students referred to at least one self-transcendent motive which is still 63%. The proportion of stable mindset students referring to *intrinsic* self-transcendent motives was 33% (8 of 24 students).

**Table 2.** Proportions, means, and total coding references within mindsets across the four motivational dimensions.

	Stable mindsets				Changed mindsets	
	High-impact	Low-impact	Social-impact	Self-impact	Low-to-social	Self-to-low
Intrinsic, self-oriented motives	51%	42%	60%	35%	57%	47%
Extrinsic, self-oriented motives	38%	49%	31%	53%	29%	46%
Intrinsic, self-transcendent motives	10%	3%	8%	4%	11%	3%
Extrinsic, self-transcendent motives	1%	6%	2%	9%	3%	4%
Total references	61	33	52	55	91	123
Mean references	10.2	5.5	8.7	9.2	10.1	8.2

*Proportions sum up to 100 within mindset; n = 6 per stable mindset; n = 9 low-to-social; n = 15 self-to-low*.

The mean number of references per mindset group was investigated as an additional inquiry to explore potential differences across mindsets. This was relevant as individual word count in the goal-setting essays has previously shown to relate to study motivation and the detailedness of the described motives per student could provide an indication of the type of mindset (Schippers et al., 2020). Accordingly, low-impact mindset students mentioned on average substantially fewer references (5.5) to their motives than the high-impact mindset (10.2 references), self-impact mindset (9.2 references), and social-impact mindset students (8.7 references).

### Typical motivational mindset cases

Four typical mindset cases were selected and described according to their motives for studying to further illustrate differences among the four mindset types.

#### Alyssa: High-impact mindset

Alyssa is a 19-year-old student with a high-impact mindset. Alyssa ascribes double meanings to her studies, including positive self-development. Yet she is also focused a lot on CV building by doing a minor program abroad, acquiring relevant knowledge, and conducting work in leadership roles. Alyssa wants to gain valuable experience to be successful in her work, while she holds an intrinsic, self-transcendent aspiration for her future career:

I would like to find out in the future how I can help other people. Perhaps it’s true that if you are successful yourself, you can better help the people around you. Who knows, you may be able to do something for the community, through a company or at least find something to serve other people with.

#### Cody: Social-impact mindset

Cody is a 20-year-old student with a social-impact mindset. He has both intrinsic and extrinsic motives for his studies. He finds studying enjoyable when it gives him a challenge while he also wants to obtain a degree to qualify for a job. It energizes him to fantasize about the future and make a concrete plan for his career. In his future work, he would love to have a good job where he can develop himself and where he feels valued. In addition, Cody has a beyond-the-self intention for his career:

I want to do something meaningful for the organization and for other people. I want to make a difference with my work and I want to do work that makes me happy.

#### Alex: Low-impact mindset

Alex is an 18-year-old student with a low-impact mindset. She aims to finish her studies because she wants to have a good job in the future. To some extent she likes to pursue her degree, because people expect this of her and she likes to fulfil this expectation. Her parents will be proud if she succeeds. Furthermore, it is important for her that she enjoys her future work and earns enough money to have a nice life. This extrinsic drive for work includes a self-transcendent orientation as she wishes to provide for her children:

Work will have to happen, but this will be a job where I will feel completely at ease. I'll make enough money to have a nice life and I want to be able to give my children everything they need. I don’t want to worry about this.

#### Robin: Self-impact mindset

Robin is an 18-year-old student with a self-impact mindset. He aims to obtain his degree because this decides the extent to which he can achieve success and have valuable experiences in his future. Robin aspires to start his own business because in this way he can build his own success. His strong focus on success is also reflected in his beyond-the-self orientation:

I hope my business becomes so successful that I can give my family some extra money and make them feel good. I want them to be proud of me.

### Changed mindsets

Table 2 and Figure 2 present the proportion of coding references *within changed mindsets* across the four motivational dimensions. To assess whether the mindset change could have already occurred during the reflection assignment, the proportion of coding references in the low-to-social-impact mindset was compared with the stable low-impact and stable social-impact mindsets. Figure 2 shows that the extent of correspondence in the low-to-social is higher with the social-impact mindset than the low-impact mindset. First, the 57% and 29% in the self-oriented dimensions of the low-to-social correspond more with the 60% and 31% in the social-impact mindset compared to the 42% and 49% in the low-impact mindset. Further, although self-transcendent motives resembled a smaller part of the coding references, particularly the proportion of 11% intrinsic self-transcendent motives in the low-to-social seems to correspond more with the 8% in the social-impact mindset compared to the 3% in the low-impact mindset. Correspondence of proportions in the extrinsic, self-transcendent dimension of motives was along the same lines albeit small: 3% in the low-to-social-impact mindset and 2% in the social-impact mindset versus 6% in the low-impact mindset.

Table 2 and Figure 2 show the extent of correspondence between the self-to-low-impact mindset and the stable low-impact mindset versus the stable self-impact mindset. The 47% and 46% in the self-oriented dimensions of the self-to-low correspond more with the 42% and 49% in the low-impact mindset compared to the 35% and 53% in the self-impact mindset. With regards to self-transcendent motives, the proportions of 3% and 4% in the self-to-low correspond more with the 3% and 6% in the low-impact mindset compared to the 4% and 9% in the self-impact mindset, although the disparities are small.

The inquiry into mean number of references was observed to further explore the extent of correspondence between the changed and stable mindset groups. Results showed that the low-to-social group provided 10.1 references on average per student. Although this surpasses the stable social-impact mindset (8.7 references), the difference is even greater compared to the stable low-impact mindset (5.5 references). The self-to-low mindset provided 8.2 references on average per student. This student group corresponds more closely with the stable self-impact mindset (9.2 references) than the stable low-impact mindset (5.5 references).

## Discussion

The current study aimed to investigate whether the goal-setting essays are indicative of a change in motivational mindset. Results showed that the motives for studying mentioned in the essays by students who changed from a low-impact mindset to a social-impact mindset correspond on all motivational dimensions more with the stable social-impact mindset than the stable low-impact mindset. Low-to-social-impact mindset students expressed intrinsic self-oriented and intrinsic self-transcendent motives while participating in the goal-setting intervention to a similar extent as social-impact mindset students. These findings suggest that the reflective assignment induced a social-impact mindset in low-impact mindset students. This provides further indications that the goal-setting intervention is beneficial for a particular type of student, namely, low-impact mindset students. Setting life goals seems to tap into the plasticity of the motivational mindset and goalsetting could indeed be regarded as a kind of *mindsetting*. Future research should probe more deeply into why it is precisely these low-impact mindset students who change their mindset. It is highly likely that these students had not yet explicated their reasons for going to university at the beginning of their study program. If students are not yet aware of their ‘why’ for university, they may be more open to new insights and can be more prone to benefit from the goal-setting intervention. The goal-setting intervention can propel students to churn to a more favorable mindset. Similarly, future research could explore why this kind of intensive reflection on life goals does not positively impact the mindset of other students. Some students may not yet be ready to commit to intrinsic reasons for their vision of the future or others simply cannot reflect sufficiently to understand their deeper desires. These students might be better helped with (one-on-one) personal career coaching or mentoring to discover their interests and explore a direction for their future (Greenfeld, 2022).

Results further showed that the self-to-low change group corresponded to a certain extent more with the stable low-impact mindset than the stable self-impact mindset in terms of extrinsic and intrinsic self-oriented motives. However, the differences in proportions were small. Moreover, motivational characteristics in the student essays of the self-to-low group corresponded closer with the stable self-impact mindset in terms of number of references per student than the stable low-impact mindset. To this end, the findings are equivocal whether the goal-setting intervention induced a ‘negative’ mindset churn in self-impact mindset students. The patterns did not show clear indications that the reflection assignment induces a less favorable mindset churn to a low-impact mindset. This lack of data suggests for now that a negative impact of the goal-setting intervention on students can be dismissed. Prior research has shown that a strong focus on extrinsic motives is debilitating for study motivation over the long term (Vansteenkiste, Lens, & Deci, 2006). Rather than the goal-setting intervention causing the churn, one could also argue that the self-impact mindset is inherently more susceptible to decrease in academic motivation based on the characteristics of the mindset. Since the design of the study where all students were expected to participate in the goal-setting intervention, we cannot rule out this alternative explanation. Future research could for instance conduct follow-up interviews with these students to unravel why they moved to a low-impact mindset during the academic year. Or an experimental design could be used similar to Morisano et al. (2010) where it could be analysed whether students in the control group show a different pattern of mindset churn or no churn at all.

Patterns in the essays of stable mindsets across the three dimensions of the MMM show similarities with the descriptions of the mindset profiles in the study by Hudig et al. (2020).^3^ Patterns in the extrinsic self-transcendent motives reveal new insight in that the self-impact mindset students seem to endorse such reasons more often than the other three mindsets. The findings further validate the MMM and the typical mindset cases enrich the four mindset profiles. Also, since almost all students (94%) had multiple motives for studying, this finding aligns with our approach to investigate these motives multidimensionally. In order to map the reality of students as well as possible, future research profits from adopting a multidimensional perspective on academic motivation.

Studies have indicated that first-year students endorse more extrinsic motives for attending university than intrinsic motives (Kennett et al., 2011). However, the proportion of coding references in our sample was larger for intrinsic motives than for extrinsic motives. The active reflection exercises in the goal-setting intervention therefore seem to evoke deeper reasons in students than a reactive online questionnaire. Self-oriented motives were generally mentioned more frequently by first-year students in this study than self-transcendent motives. However, contrary to studies indicating that business education fosters selfish motives (Miller & Xu, 2016), results did indicate that two-thirds of stable mindset students seemed to have an orientation to not only benefit themselves. A third of these students had the more beneficial *intrinsic* self-transcendent motives for studying. This points to the value that guided reflection on life goals as part of a curriculum can have in drawing out prosocial motives. Yeager et al. (2014) asked students to reflect and write about how the world could be a better place which bolstered an intrinsic beyond-the-self orientation in students. The goal-setting intervention might integrate such an exercise to direct students’ thinking more strongly towards intrinsic self-transcendent reasons for their goals. Recent research also showed that students express self-transcendent motives in writing much less in comparison to face-to-face interviews (Kuusisto & Schutte, 2022). Hence, follow-up interviews could provide complementary insight into students’ motivational mindset but also the working dynamics of the goal-setting intervention.

### Strengths, limitations, and future research

This exploratory qualitative study has both strengths and limitations. A key limitation is the small sample sizes in the stable mindsets and the changed mindsets. Due to these small sample sizes as well as the qualitative nature of the study, we avoided statistical analyses to test whether the coding references in the mindset groups differed significantly. Furthermore, participants in the two changed mindset groups were not randomly assigned and the groups seemed to have an a-typical distribution in terms of gender compared to the stable mindsets. Additionally, we used business education students and the findings are therefore not generalizable to other populations. Future research could further investigate the mindsets in the context of the goal-setting intervention in larger and more diverse samples. Importantly, given the research design of this study we can only provide indications and we cannot make any claims of causal evidence. Future research could conduct an experimental study with an active control group to further explore the proposed working mechanism of the goal-setting intervention. The design of the goal-setting intervention was also not set up to measure students’ motives for studying. Hence, challenges were posed onto the coders to extract the goal motives due to ambiguous and implicit writing of students. It is demanding for students to explicate one’s thoughts into writing and a concern may be that the questions focus on students’ ability to articulate a response and not their underlying motives (Geer, 1988). Ambiguous language was particularly present in the intrinsic and extrinsic, self-oriented dimensions. Future research on the goal-setting intervention could integrate tick-box questions to extrapolate the study- and work-related motives for students’ life goals more explicitly, with inspiration from existing questionnaires such as the questionnaire used by Heather Malin (2022) and other colleagues (e.g., Roberts & Robins, 2000). Future research could also complement open-ended questions with in-depth interviews to address this potential issue. On a similar note, we cannot exclude that social desirability was influential in the student essays as students might have generated certain motives out of compliance to the school assignment. However, since the assignment was delivered solely for personal benefit and students had not to disclose the answers of the essay to their tutor, we deem the influence of this factor in the essays small. As the content of the motives was outside the scope of this study, future research could conduct a thematic analysis on the motivational dimensions within the essays to further explore the mechanisms of the goal-setting intervention. We also had to focus on study- and work-related goal motives due to the aims and scope of this study. Future research might explore the motivational mindsets in the context of all life goal domains.

Despite these limitations, there are several strengths worth highlighting. Building on recent quantitative research (Hudig et al., 2022), this qualitative study included an intensive and rigorous collaboration between coders to derive life goal motives from essays of first-year students. As a result of our qualitative approach, the development of the MMM was advanced and the characteristics of the mindset profiles were enhanced. The present paper points towards the multidimensional nature of academic motivation and contributes to a better understanding of the reality of students in higher education. Most importantly, exploring student essays in a deductive manner was worthwhile to further excavate the working mechanism of the goal-setting intervention. Having a better understanding of this mechanism is vital as the goal-setting intervention has shown to improve study success particularly among potential struggling students (Morisano et al., 2010; Schippers et al., 2020). The findings of this paper encourage researchers and practitioners to focus on the ‘why’ of university for students and promote the implementation of life goalsetting in more relevant settings.

## Conclusions

The first year of the study program is crucial for students’ study career and the goal-setting intervention has proven to boost students’ first-year study success. The psychological processes that drive these positive effects have so far remained elusive. This qualitative study builds on recent findings in which the underlying mechanism of the goal-setting intervention was proposed. An investigation into the written goal-setting essays substantiated the indications that students who enter university with a low-impact mindset cultivate intrinsic motives for studying during the guided reflection exercises and change to a social-impact mindset. We could regard goalsetting therefore as a kind of mindsetting because it taps into the plasticity of students’ motivational mindset. Low-impact mindset students have shown to be an at-risk group for first-year dropout, and the present study further illustrates that these students benefit most from the goal-setting intervention. The findings in this study did not substantiate the indications that students may change to a low-impact mindset as a result of the goal-setting intervention. Future research has to investigate why students adopt a low-impact mindset over the course of the first academic year. The goal-setting intervention is a powerful tool to help students reflect on a deeper level on their lives and the why of their university education. This study serves as a basis for better understanding for whom, why and when the goal-setting intervention works, which is useful for higher education institutions to support more students succeed in their studies and foster their wellbeing and sense of purpose in life.

## Notes

1. Randomly selected student essays from prior research that were not included in the present study.

2. Idem.

3. Intrinsic self-oriented, intrinsic self-transcendent, and extrinsic self-oriented.
